# Comparative Proteomic Analysis of Different Parts of *Taenia Hydatigena*

**DOI:** 10.3389/fvets.2021.626579

**Published:** 2021-04-26

**Authors:** Mengting Cai, Yating Li, Guitian He, Xiaola Guo, Shaohua Zhang, Lujun Yan, Jing Zhang, Juntao Ding

**Affiliations:** ^1^College of Life Science and Technology, Xinjiang University, Urumqi, China; ^2^State Key Laboratory of Veterinary Etiological Biology, Key Laboratory of Veterinary Parasitology of Gansu Province, Lanzhou Veterinary Research Institute, Chinese Academy of Agricultural Sciences, Lanzhou, China; ^3^National Institute of Parasitic Diseases, Chinese Center for Disease Control and 4 Prevention, Key Laboratory of Parasite and Vector Biology, Ministry of Health, National Center for International Research on Tropical Diseases, World Health Organization Collaborating Center for Tropical Diseases, Shanghai, China

**Keywords:** *Taenia hydatigena*, scolex, cyst, cestode, proteome

## Abstract

*Taenia hydatigena*, a globally distributed parasite, is a canine tapeworm and causes huge economic losses in the food industry. Using LC-MS/MS, the proteomes of *T. hydatigena* cyst scolex, designated as CS, and the cyst without the scolex, designated as CWS, were profiled and a total of 764 different proteins were identified, 664 of which were identified in CS, 412 identified in CWS, and 312 in both. Comparative analysis revealed that CS had more abundant proteins associated with growth and development, while CWS had more abundant proteins constituting a scaffolding and protective extracellular matrix. Consistent with the sequencing data, the abundance of the five selected proteins was validated to be higher in CWS than CS by Western blotting. The current data will provide a clue for further pinpointing a role of these proteins in the biology of *T. hydatigena*.

## Introduction

*Taenia hydatigena* is a tapeworm residing in the small intestine of canines and is widely distributed across the world. Infection of *T. hydatigena* larvae is extensively found in sheep, goats, and pigs but rarely in cattle and wild ruminant animals. The disease by *T. hydatigena* larvae is serious and even lethal in lambs and piglets, and it is one of the causes of huge economic losses in the food industry (1).

To complete the entire life cycle, *T. hydatigena* needs two different animals. The adults reside in the intestine of definitive hosts such as dogs, wolves, and foxes, whereas the larvae mainly parasitize the liver serosa and mesentery of intermediate hosts such as pigs, sheep, and goats. The intermediate hosts are usually infected upon digestion of food or water contaminated with eggs that are expelled with the feces by infected canines. Following egg entry into the host digestive tracts, the oncospheres are activated and then migrate with the blood through the liver to intestinal momentum for further development into *Cysticercus tenicollis*, which contains transparent liquid. If canines consume *C. tenicollis*-infected offal or organs, the larvae develop into adult worms in their intestine, thus completing its life cycle (2, 3).

In general, the protein components of different parts/tissues in a given organism vary greatly, and the discrepancy is largely attributed to the physical and functional differences. Therefore, proteomic data of the different parts of *T. hydatigena* is helpful for us to profoundly understand the biological characteristics of *T. hydatigena*. For instance, the proteomic analysis of *T. hydatigena* cyst fluid found that there were a plethora of proteins participating in the amino acid synthesis and complement cascades, suggesting unique microenvironment of the cyst (2, 3). Up to now, the differences in the protein constitution of other parts of the *T. hydatigena* metacestode, such as the scolex and cyst wall, have still been unclear.

Using high performance liquid chromatography-coupled tandem mass spectrometry (LC-MS/MS) technology, the proteomes of two different parts of *T. hydatigena*, the scolex and the rest part of the larvae, were comparatively defined. Moreover, the expression levels of five selected proteins in these two parts were further validated by Western blotting. The current data will provide a clue for investigation of biological functions of these proteins in future studies.

## Materials and Methods

### Isolation of *C. tenuicollis* and Preparation of Proteins

**Figure 1:**
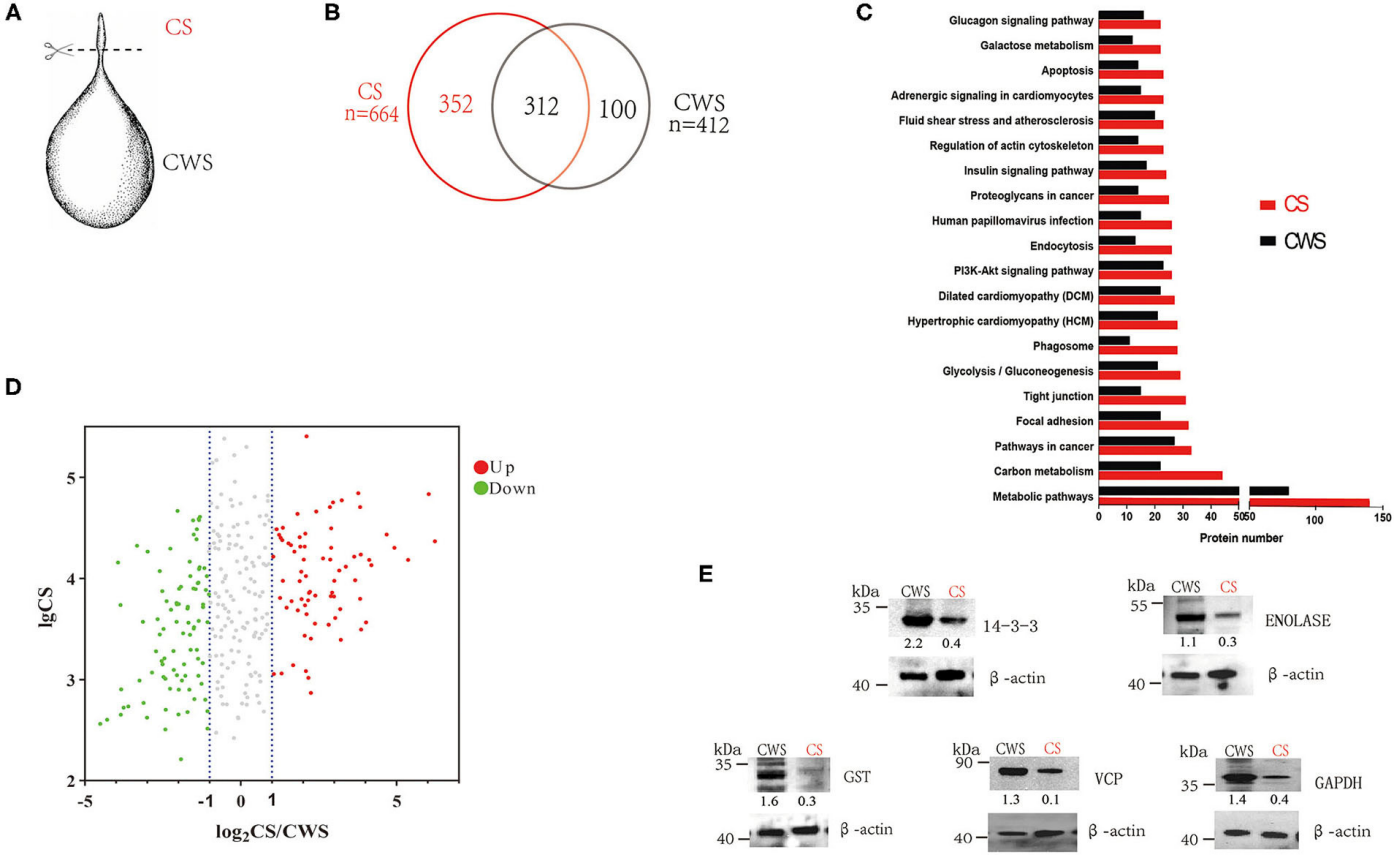
Comparative proteomes of the scolex and the rest part of *T. hydatigena* cyst. **(A)** Individual *T. hydatigena* cysts were scissored into two parts, the scolex, designated as CS, and the rest (the cyst without scolex), designated as CWS. **(B)** Protein identification in different two parts. (**C**) Top 20 pathways in CS and CWS proteins. **(D)** 312 proteins commonly shared by CS and CWS. Up- and down-expressed protein in the CS were identified with an abundance fold change (CS/CWS) of 2 and 0.5, respectively. To clearly show which proteins are abundant and differentially expressed, a volcano plot was pictured by use of CS_abundance_ and abundance fold changes. **(E)** Validation of 5 proteins by Western blotting. Numbers represent relative abundance of a given protein in CS or CWS. Beta actin is used as a reference and its abundance is set as 1.

Three fresh *C. tenuicollis* samples were carefully dissected from the mesentery of slaughtered adult sheep in an abattoir, Xingjiang Autonomous Region, China. After five washes with sterile and ice-cold PBS, individual cysts were scissored into two parts, the scolex, designated as CS, and the rest (the cyst without scolex), designated as CWS (Figure 1A).

Afterward, 50 mg of CS and CWS were promptly ground into powder with a mortar in liquid nitrogen, respectively, followed by the addition of protease inhibitor cocktail (Sigma) and then agitation overnight at 4°C. Samples were centrifuged at 12,000 g for 20 min at 4°C, followed by sterilization using 0.22 μm filters (Millipore). The protein concentration was determined using Bradford (Beyotime). Protein samples were directly used or stored at −80°C.

### Label-Free Quantitative Proteomics

The protein preparations were separated by gel electrophoresis, and then protein strips were captured at different locations and separately processed. The gel was enzymatically hydrolyzed, and the peptides were extracted using by a Shimadzu LC-20AD model nanoliter liquid chromatograph. The peptides were then analyzed using LC-MS/MS as previously described (4). Briefly, the separated peptides were ionized by a nanoESI source and then passed to a tandem mass spectrometer LTQ Orbitrap Velos (ThermoFisher) for data-dependent acquisition mode detection. The main parameters were set as follows: the ion source voltage was set to 2.2 kV; the MS_1_ scan range was 350 ~1500 m/z; the resolution was set to 30,000; the MS_2_ starting m/z was fixed at 100; the resolution was 7,500. The screening conditions for the MS_2_ fragmentation were charge 2+, 3+, and 4+ or higher, and the top eight parent ions with the peak intensity exceeding 1,000. The ion fragmentation mode was higher energy collisional dissociation (HCD) with normalized collision energy (NCE) set to 35, and the fragment ions were detected in Orbitrap. The dynamic exclusion time was set to 15 s. In this pipeline, the results from search engine were pre-processed and re-scored using Percolator to improve the matching accuracy (5). The output was then filtered by false discover rate (FDR, ≤ 0.01) at a spectral level to obtain a significant identified spectrum and peptide list. Then, based on the parsimony principle, we performed protein inference on peptides and generated a series of protein groups. In order to obtain the abundance of each protein, this pipeline used BGI's own software to complete the extraction of peptide extracted ion chromatograms and calculate the peak area. Then, based on the intensity-based absolute-protein-quantification (iBAQ) algorithm, the total peak area in each protein group was divided by the number of theoretical peptides to obtain the final abundance of each protein. Due to the unavailability of the *T. hydatigena* genome, the protein data were searched using Mascot (Matrix Science, version 2.3.03) against *Taenia solium* protein database retrieved from Gene DB (12,329 sequences, http://www.genedb.org/Homepage/Tsolium) with parameters as previously completed (6, 7). Protein identification was conducted as previously described (8). Gene Ontology (GO) terms (http://geneontology.org/) and Kyoto Encyclopedia of Genes and Genomes (KEGG) pathways (https://www.genome.jp/kegg/) were used to comparatively analyze the identified proteins.

### Western Blotting

A total of 20μg of CS and CWS samples were subject to 10% SDS-PAGE gel electrophoresis, respectively, and then transferred onto polyvinylidene fluoride membranes (Millipore). After incubation with 5% bovine serum albumin (Sigma), the membranes were treated overnight at 4°C with rabbit anti-beta Actin (1:1000, Abcam), rabbit anti-GST (1:1000, Life Science Products & Services), rabbit anti-GAPDH (1:1000, Sigma), rabbit anti-14-3-3 (1:1000) (9), mouse anti-Enolase (1:1000) (9), and rabbit anti-Transitional endoplasmic reticulum ATPase (VCP) (1:1000, previously prepared in our lab), respectively. Then the membranes were incubated for 1 h at room temperature with corresponding HRP-conjugated antibody: goat anti-mouse IgG antibody (1:10,000, SeraCare Life Sciences) or goat anti-rabbit IgG antibody (1:10,000, SeraCare Life Sciences). Finally, signals were visualized on X-ray films using enhanced chemiluminescence (ThermoFisher).

## Results

In CS, a total of 37,726 spectra were obtained, leading to identification of 2,359 peptides and 664 proteins (Supplementary Table 1). In CWS, a total of 38,166 spectra were obtained, leading to identification of 1,433 peptides and 412 proteins (Supplementary Table 2). In total, 764 non-redundant proteins were identified from both CS and CWS datasets, 40.8% of which were commonly shared by two samples. A total of 352 proteins and 100 proteins were exclusively present in CS and CWS, respectively, and 312 in both (Figure 1B). Among the shared proteins, 73 proteins were up-regulated, while 89 were down-regulated in CS compared with CWS (Figure 1D). Approximately 91.7% of 664 CS proteins were annotated into 296 different pathways (Supplementary Table 3), while 88.6% of 412 CWS proteins annotated into 271 different pathways (Supplementary Table 4). Among them, metabolic and carbon metabolism and focal adhesion pathways were highly represented in both CS and CWS (Figure 1C). GO analysis showed that 209 (31%) CS and 108 (26%) CWS proteins were predicted to participate in the metabolic process.

Nine proteins that were highly represented in CS were also found to be abundant in CWS, including paramyosin, gelsolin, myosin heavy chain, fructose 1,6 bisphosphate aldolase, basement membrane specific heparan sulfate, spectrin alpha chain, tropomyosin, and filamin (Table 1). Moreover, there was the presence of a variety of collagen-associated proteins, especially in CWS, including collagen alpha 1 (V) chain, collagen alpha 2 (I) chain, collagen type XI alpha 2, collagen B type II, and fibrillar collagen chain FAp1 alpha. Furthermore, collagen or related proteins were much more abundant in CSW than CS (Table 1).

**Table 1:** Top 20 proteins abundant in both CS and CWS.

**Protein**	**Size (kDa)**	**CS**		**CWS**		**Protein ID**
		**Unique peptide**	**Unique spectra**	**Unique peptide**	**Unique spectra**	
Paramyosin	107.7	45	179	37	151	TsM_001115200
Myosin heavy chain	225.2	56	144	30	84	TsM_000676900
Basement membrane specific heparan sulfate	881.2	67	125	72	153	TsM_000123600
Fructose 1,6 bisphosphate aldolase	39.7	12	80	15	184	TsM_000467100
Enolase	46.5	12	60	11	60	TsM_000595600
Spectrin alpha chain	130.7	33	55	24	45	TsM_000861200
Heat shock 70 kDa protein 4	71.1	12	55	9	36	TsM_001208400
Spectrin beta chain brain 3	159.6	27	53	16	33	TsM_000526200
14-3-3 protein	28.1	10	44	8	33	TsM_000719200
Basement membrane specific heparan sulfate	93.4	19	43	19	49	TsM_001048700
Filamin	129.9	22	36	21	53	TsM_001158800
Filamin	182.3	15	34	20	54	TsM_000523500
Collagen alpha 1(V) chain	231.6	14	32	13	33	TsM_000925000
Phosphoglycerate kinase 1	42.3	11	31	3	3	TsM_000796500
Phosphoenolpyruvate carboxykinase	70.2	29	120	/	/	TsM_000763700
Glyceraldehyde 3 phosphate dehydrogenase	36.2	11	62	/	/	TsM_000056400
Cytosolic malate dehydrogenase	36.5	11	48	/	/	TsM_000048200
Glycogen phosphorylase	73.3	15	46	/	/	TsM_000970200
14-3-3 protein beta:alpha	27.7	8	32	/	/	TsM_000747500
Glucose 6 phosphate isomerase	61.7	11	36	/	/	TsM_000412200
Collagen alpha 1(V) chain	165.5	/	/	14	53	TsM_000649300
Filamin-c isoform g	117.6	/	/	24	51	TsM_000009200
Delta aminolevulinic acid dehydratase	39	/	/	8	50	TsM_000228100
Type II collagen B	127	/	/	11	43	TsM_000554300
Fibrillar collagen chain FAp1 alpha	130	/	/	7	36	TsM_000554400
Phosphoglycerate mutase	28.5	/	/	9	34	TsM_001053100

‘*/': not applicable*.

According to the difference of protein abundance (Table 1), 14-3-3, enolase, glutathione S-transferases (GST), VCP, and glyceraldehyde-3-phosphate dehydrogenase (GAPDH) were selected and verified by Western blotting. Consistent with the proteomics data, the results showed that the expression of all the selected proteins was higher in CWS than that in CS (Figure 1E).

## Discussion

Among the proteins identified in the study, the 14-3-3 protein, a plasmon protein that binds to signaling proteins, is involved in the regulation of parasite growth and development. Moreover, it is a promising vaccine antigen and vaccines based on 14-3-3 have been utilized to fight against parasitic diseases (10, 11). Therefore, the expression of 14-3-3 in both CS and CWS makes it a promising vaccine candidate or drug target for control of *T. hydatigena* infection.

In the study a number of proteins were found to be abundant in both CWS and CS, including paramyosin. Paramyosin, also called as B antigen in helminthes, is a 97 kDa myofibrillar protein with a coiled-coil structure found only in invertebrates and is capable of inducing strong anti-infective immune protection, thus being the most promising vaccine candidate antigen (12). It was found that anti-paramyosin monoclonal antibody conferred resistance to infection with *Schistosoma japonicum* in mice (13), and recombinant paramyosin protected water buffalo from *S. japonicum* infection (14). Moreover, paramyosin was also found to be an immunodominant antigen and induce highly protective immune responses against T. *solium* (15, 16). Therefore, the high expression of paramyosin in the whole body may render it as a promising drug target or vaccine antigen against *T. hydatigena*.

Like paramyosin, the enzyme fructose 1, 6 bisphosphate aldolase was also abundant in CWS and CS. This enzyme is involved in energy production in the glycolysis and is also associated with many non-glycolysis functions in parasites, such as adhesion to host cells, plasminogen binding, and invasion (17). Previous studies have demonstrated that parasite-derived aldolase interacts with invasin proteins of *Toxoplasma gondii*, and then directs the entry and motility of parasites by binding to the cytoplasmic tail of the micronemal protein 2 (18). Whether the high expression of fructose 1, 6 bisphosphate aldolase is beneficial to parasite infection needs to be investigated in future.

The current study also found more collagen-associated proteins in CWS than CS. Collagen is the most abundant protein in animals, accounting for more than 30% of the total proteins of animal tissues, and is closely related to organ functions (19). In addition, it is also thought to be a common structural protein with the antigenicity (20). Although their functions in parasites remains unclear, the existence of many types of collagen components may be explained by that they constitute a complex scaffolding and protective extracellular matrix in CWS.

Conversely, phosphoenolpyruvate carboxykinase (PEPCK), a key enzyme in carbon metabolism, was more enriched in CS than CWS (Table 1). PEPCK was also found to be abundant in *T. hydatigena* cyst fluid (2, 3) and *Echinococcus granulosus* cyst fluid (8). Similarly, another enzyme in carbon metabolism, phosphoglycerate kinase 1, was also more enriched in CS than CWS, which was abundant in *T. hydatigena* cyst fluid (2, 3) but not in *E. granulosus* cyst fluid (8). Of them, PEPCK is involved in the glycolysis in the cestodes, but it carries out a gluconeogenic role in hosts. Because of the difference in primary functions, this enzyme is regarded as a plausible anthelmintic target (21). Recently, PEPCK was shown to exhibit the highest activity in *Leishmania donovani* under glucose starvation, facilitating the growth and survival in the hostile environment possibly via gluconeogenesis (22). Higher abundance of PEPCK suggests that the sugar metabolism is an important event in the scloex, and in-depth studies on this enzyme help us to further understand its role in development.

## Conclusion

The current study reveals the different protein constitution between CS and CWS. CS had abundant enzymes involving in metabolism, such as cytosolic malate dehydrogenase, phosphoglycerate kinase 1, and PEPCK, which may be associated with the growth and development. Unlike CS, CWS had more abundant proteins constituting a scaffolding and protective extracellular matrix, such as collagen alpha 1(V) chain. The data will provide useful references to further investigate the role of those proteins in *T. hydatigena* biology.

## Data Availability Statement

The datasets presented in this study can be found in online repositories. The names of the repository/repositories and accession numbers can be found below: The mass spectrometry proteomics data have been deposited to the ProteomeXchange Consortium via the PRIDE (1) partner repository with the dataset identifier PXD023543.

## Author Contributions

JD and JZ conceived the study. MC, YL, GH, XG, and LY conducted the experiments. YL, MC, SZ, and XG analyzed the data. MC, YL, and JD wrote the paper. All authors contributed to the article and approved the submitted version.

## Conflict of Interest

The authors declare tha the research was conducted in the absence of any commercial or financial relationships that could be construed as a potential conflict of interest. The reviewer JD declared a shared affiliation with the authors, GXL, ZSH, to the handling editor at time of review.
